# Diminazene aceturate or losartan ameliorates the functional, radiological and histopathological alterations in knee osteoarthritis rodent model: repurposing of the ACE2/Ang1-7/MasR cascade

**DOI:** 10.1186/s40634-023-00673-1

**Published:** 2023-10-25

**Authors:** Yasser H. Habib, Eman Sheta, Mahmoud Khattab, Mennatallah A. Gowayed

**Affiliations:** 1 Department of Pharmacology and Toxicology, Faculty of Pharmacy, Cairo University, Cairo, Egypt; 2 Department of Pathology, Faculty of Medicine, Alexandria University, Alexandria, Egypt; 3 Department of Pharmacology and Therapeutics, Faculty of Pharmacy, Pharos University in Alexandria, Canal El- Mahmoudia Str., Smouha, Alexandria, Egypt

**Keywords:** Osteoarthritis, Oxidative stress, Inflammatory mediators, RAAS, Diminazene aceturate, Losartan

## Abstract

**Purpose:**

Current therapies for osteoarthritis (OA) are limited to analgesics and anti-inflammatory drugs. Considering the importance of oxidative stress and inflammatory mediators in OA etiology, we tested the hypothesis that targeting the renin–angiotensin–aldosterone system (RAAS) can improve OA anomalies. Diminazene (DIZE), an activator of angiotensin-converting enzyme 2 and the angiotensin 2 type-1 receptor blocker losartan (LOS) were used for this purpose.

**Methods:**

OA was induced by a single intra-articular injection of monosodium iodoacetate. The effects of exposure to DIZE or LOS for 21 days on OA anomalies in rats’ knees were investigated. Evaluation of motor function, nociception, and inflammatory response was done using rotarod, knee bend and knee swelling tests. Markers of knee joint inflammation, and cellular oxidation in addition to the RAAS biomarkers, were assessed in knee tissues, along with radiological and histopathological investigations.

**Results:**

Elevations in inflammatory and oxidative markers in knee tissues of OA rats were mostly improved by the two therapeutic drugs. Such effect was also reflected in the rotarod, knee bend and knee swelling tests. Treatment with DIZE has shown a more prominent effect than LOS in controlling OA-associated inflammation and cellular oxidation. Markers of RAAS have also shown better responsiveness to DIZE over LOS.

**Conclusions:**

DIZE has shown a prominent increase in the angiotensin 1–7 amount, highlighting the involvement of the signaling pathway in the immunomodulatory effect. The radiological and histopathology examination came to confirm the outcome of biochemical markers, nominating diminazene aceturate as a possible therapeutic option for OA.

**Graphical Abstract:**

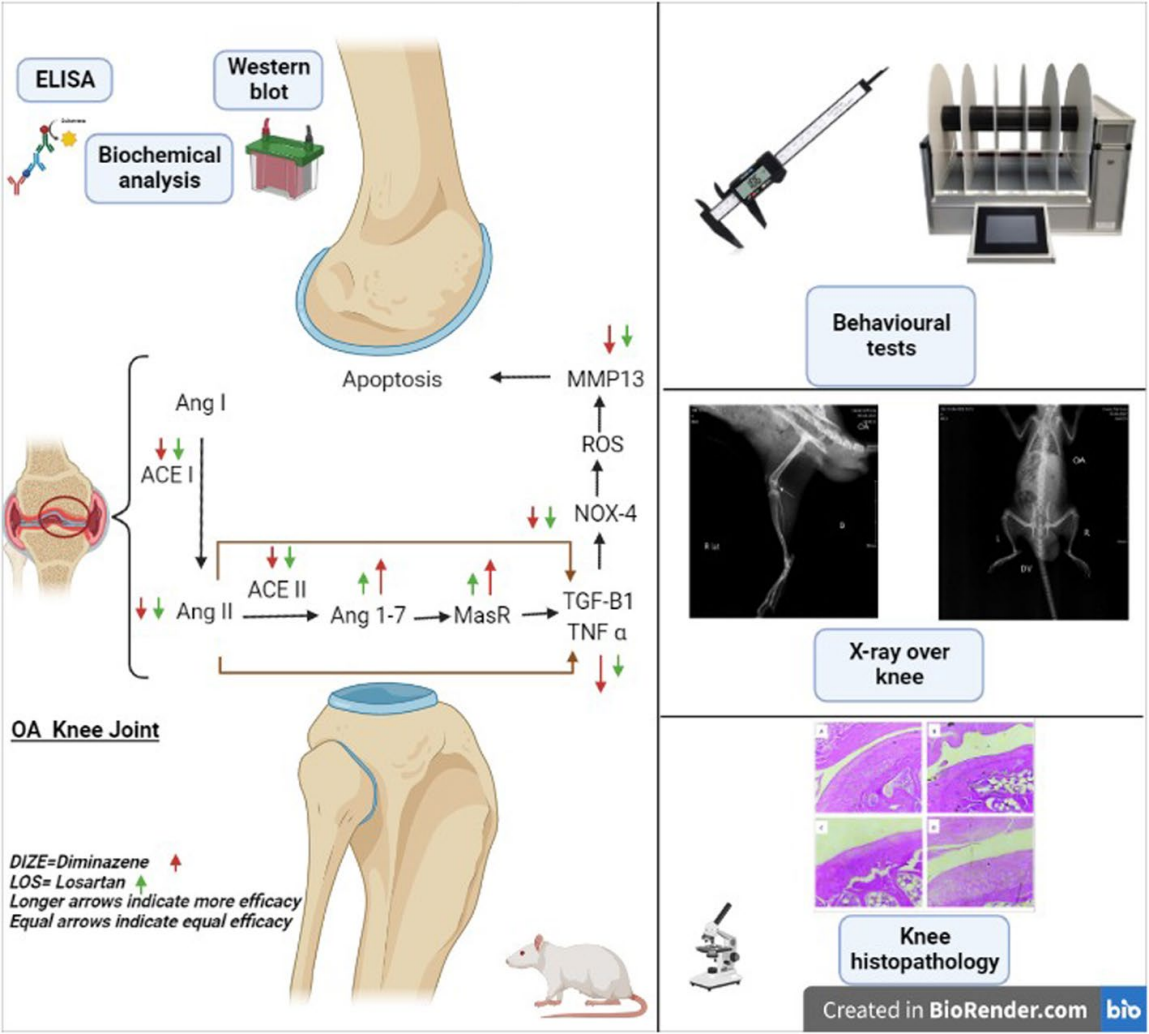

**Supplementary Information:**

The online version contains supplementary material available at 10.1186/s40634-023-00673-1.

## Introduction

Osteoarthritis (OA) is a chronic musculoskeletal disorder that leads to structural and functional failure of synovial joints [7, 30]. It is characterized by severe pain resulting from the loss of articular cartilage, followed by several consequences such as subchondral bone remodeling, formation of osteophytes, development of bone marrow lesions and changes in the structure of joint components such as synovium, joint capsule, and ligaments [36].

Oxidative stress and reactive oxygen species (ROS) play a crucial role in the development of OA. The nicotinamide adenine dinucleotide phosphate (NADPH) oxidase enzymes are major contributors to the development of ROS in the synovial fluid. Upon activation, a fully active enzyme system develops in the plasma membrane, which in turn generates ROS in the chondrocytes contributing to chondrocyte apoptosis, extracellular matrix (ECM) breakdown and the generation of cytokines [59]. Lately, NADPH oxidase-4 (NOX-4) has shown main involvement in the production of ROS and pro-inflammatory cytokines in chondrocytes, promoting degradation of articular ECM, and triggering the development of OA [21, 22, 25].

The renin–angiotensin–aldosterone system (RAAS) has been involved in several diseases' pathology by increased expression of NADPH oxidase and the associated ROS production [9, 31, 32]. Angiotensin (Ang) II is a member of classic RAAS and a prooxidant peptide as it promotes ROS production through the activation of membrane-bound NADPH oxidase [12]. The resulting molecules are involved in vasoconstriction and vascular hypertrophy in vascular smooth muscle cells [58]. On the other hand, angiotensin-converting enzyme 2 (ACE2) catalyzes the conversion of the vasoconstrictor and pro-oxidative peptide Ang II to vasodilatory and antioxidative metabolite Ang-(1–7), which have an important role in the protection of the heart and its vessels by interacting with G-protein-coupled Mas Receptor (MasR) [43].

In addition to the circulating RAAS, local tissue RAAS is expressed in various tissues exerting diverse physiological effects [34]. Main components of tissue-based RAAS, including ACE/Ang II/AT-1R have been found to be synthesized and active in bone cells, osteoblasts and osteoclasts [13, 15]. The counterregulatory role of ACE2/Ang 1–7/MasR against the classical ACE/Ang II/ AT_1_R axis has been well-studied in cardiac physiology and in the pathophysiology of heart failure [33]. The role of the two RAAS cascades is much less studied outside the cardiovascular system. ACE inhibitors and angiotensin receptor blockers (ARBs) are clinically approved drugs known to enhance ACE2 mRNA expression, protein levels and/or activity [4].

Animal experimental and clinical human evidence indicates the potential role of RAAS as a target in arthritis and bone health. Chondrocytes from arthritis patients were shown to express both AT-1R/AT-2R mRNA and proteins [17, 54]. Furthermore, RAAS components were upregulated in synovial tissues from collagen-induced arthritic rats and from arthritis patients [54, 55]. Serum renin and ACE levels were similar in arthritis patients and in healthy controls in contrast with higher levels in synovial fluid of arthritis patients [6]. The expression of the main RAAS components in the trabecular bone of lumbar vertebrae in patients suffering from glucocorticoid-induced osteoporosis was higher than in the control and was negatively associated with bone mineral density [46]. In the last ten years, research in animals demonstrated beneficial effects for the activation of ACE2/Ang1-7 in bone diseases [2, 3].

Diminazene aceturate (DIZE) is an antitrypanosomal agent that gained attention in the last years due to its therapeutic potential in multiple diseases [29, 37, 44, 60]. DIZE has shown to be a main activator of ACE2 [53], having a similar chemical structure to the recognized ACE2 activator xanthenone [10]. An earlier study confirmed that DIZE increases MasR expression as well as ACE2 levels, showing a correlation between DIZE/ACE2 and the induction of Ang (1–7)/Mas axis expression [47]. It also inhibited cytokine production and decreased the expression of Ang II and AT-1R, while increasing Ang (1–7) expression in human retinal pigment epithelium cells [49].

Despite being an immunomodulatory agent with antioxidant and anti-inflammatory potential [29, 60], no previous studies explored the therapeutic potential of DIZE in osteoarthritis. Hence, the aim of the current study was to test the ability of DIZE to tone down joint anomalies in an osteoarthritis rat model, test the applicability of repurposing the ACE2/Ang1-7/MasR cascade activation in OA and compare its effectiveness to the ARB, losartan. The anti-inflammatory and antioxidative stress mechanisms were monitored together with the tissue RAAS biomarkers.

## Materials and methods

### Animals

Adult male Sprague–Dawley rats weighing 170–200 gm were purchased from the animal house of the University. Five male rats per cage were kept under observation in a standard environmental condition of 23°C–25 °C, 12 h light/dark cycle preceding the study with free access to water and a standard chow diet. All the animal manipulations and care were performed as instructed by the national guidelines for the care and use of laboratory animals [49] and in accordance with the “Research Ethical Committee” of the local institution (Approval No.: PO.3.4.4). The study complies also with ARRIVE guidelines and the “National Research Council’s Guide for the Care and Use of Laboratory Animals”.

### Drugs and chemicals

Monosodium iodoacetate (Sigma-Aldrich, St. Louis. MO, USA, cat no. I2512), Diminazene aceturate (Sigma-Aldrich, St. Louis, MO, USA) and ketamine/xylazine (Biochemie GmbH, Vienna, Austria) were purchased from commercial suppliers. Losartan was a gift from Amriya for Pharmaceutical industries, Alexandria, Egypt. All drugs were dissolved in saline.

### Induction of osteoarthritis (OA)

Osteoarthritis experimental rat model was induced through a single intra-​articular injection of monosodium iodoacetate (MIA, 3 mg/50 μL) dissolved in sterile saline, into a shaved and disinfected knee joint [14]. The rat was anesthetized using an intraperitoneal mixture of ketamine/xylazine (50/5 mg/kg) before injection [61]. The OA was induced always in the right knee joint. Chronic inflammation was allowed to progress in all animals for 20 days [14].

### Experimental design

Osteoarthritic rats were distributed randomly into three groups of ten rats each: (i) OA/saline orally, (ii) OA/Diminazene intraperitoneal (DIZE, 15 mg/kg/day) [5]. (iii) OA/losartan orally (LOS, 20 mg/kg/day) [40], in addition to (iv) normal healthy rats’ group (CN) taking saline orally. Drugs were administered starting from day 20 after induction for 21 days. Coordination, balance and inflammation were assessed for adult rats via rotarod, knee bent, and knee swelling tests. At the end of the study, rats were euthanized using an overdose of phenobarbital (200 mg/kg i.p.), and the injected knee joints were isolated and stored at -80°C for ELISA & western blot analysis. Four knee samples of the injected knee joints from each group were fixed in 10% formaldehyde and embedded in paraffin blocks for histopathological investigation.

### Behavioral tests

#### Rotarod test

The functional motor activity including coordination and balance were assessed by a rotating rod. To create forced motor activity animals were trained to stay on the rotarod for 5 min with a speed of 20rpm. The training was applied for 3 consecutive days one week before induction. Then the test was done on day zero before induction and day 41 before the sacrifice with a speed of 20 rpm until falling. Rats were allowed to rest for 15 min between each measurement. The latency to falling was recorded for 3 measurements, the average was calculated and used for statistical analysis [14, 35]. The percentage of change in locomotive performance from day zero was calculated.

#### Knee bend test

This test was carried out to assess OA-associated knee joint pain. The injected knee joint was subjected to five alternate flexions and extensions. The animal squeaks and/or struggles were counted and scored as follows: 0 = no response to joint movement, 0.5 = struggle to maximal flexion or extension, 1 = struggle to moderate flexion or extension/ vocalization to maximal flexion or extension, 2 = vocalization to moderate flexion or extension. The test was performed before and after treatment (days 20 and 41). The sum of all scores was calculated as an indication of the grade of nociception [14].

#### Knee joint swelling

This test was carried out to assess OA-associated knee joint inflammation. On day 42, the end of the experiment, rats were sacrificed and the skin around the knee was opened. The knee diameter was measured by a caliper as an indicator of inflammation and edema. The difference in knee diameter was calculated in mm using the vernier caliper [14].

### Enzyme-Linked Immunosorbent Assay (ELISA) measurement of key knee joint biomarkers

Assessment of RAS biomarkers was performed by measuring the knee joint content of Angiotensin-converting Enzyme 1 (ACE1, Cat # MBS733102), Angiotensin-converting enzyme 2 (ACE2, Cat # MBS764117), Angiotensin 1–7 (Ang 1–7, Cat # MBS2604372) and Angiotensin II (Ang II, Cat # MBS705139). For the assessment of the inflammatory biomarkers, the knee joint content of transforming growth factor beta (TGF-β1, Cat # MBS824788) was measured. Assessment of oxidative stress biomarkers was done by measuring the knee joint content of nicotinamide adenine dinucleotide phosphate oxidase 4 (NOX-4, Cat # MBS2503069).

The level of each biomarker was determined in knee tissue homogenates using ELISA kits (myBioSource, CA, USA) according to the manufacturer’s instructions. The detailed ELISA procedure is available as Additional file 1 to this manuscript.

### Western blot

The articular cartilage of the rats at the knee joint was excised carefully after sacrificing under a pentobarbital overdose (200 mg/kg) [20]. The excised cartilage was cut into thin sections and then homogenized in the Laemmli sample buffer 4% SDS, 10% 2-mercaptoethanol, 20% glycerol, 0.004% bromophenol blue and 0.125 M Tris HCl. The pH was checked and brought to 6.8. Each previous mixture was boiled at 95°C for 5 min to ensure denaturation of protein before loading on polyacrylamide gel electrophoresis. The concentration of proteins in the supernatant was measured by Bradford Protein Assay Kit (SK3041, Bio basic Inc., Markham Ontario, Canada) for quantitative protein analysis. The protein samples (20–30 μg) were resolved by electrophoresis on 8–10% sodium dodecyl sulfate–polyacrylamide gel (SDS-PAGE) and subsequently electroblotted onto polyvinylidene fluoride (PVDF) membranes. The membrane blocking was achieved by incubation with 3% bovine serum albumin tris-buffered saline with Tween 20 (TBST) buffer at room temperature for 1 h. The incubation of membranes with primary antibodies was carried out overnight at 4 °C. The antibodies used were anti-MasR (cell signaling technology, MA, USA, Cat #3707), anti-TGF-β1 (Santa Cruz Biotechnology, CA, USA, Cat # G-1: sc-390453), and anti-MMP-13 (Santa Cruz Biotechnology, CA, USA, Cat # C-3: sc-515284). Incubation was carried out at room temperature with HRP-conjugated secondary antibodies for 1 h. The enhanced chemiluminescence detection system was employed for visualization of the bands and the ChemiDoc MP imager (Media Cybernetics, Bio-Rad's, INC, Canada) was used to read the band intensity of the target proteins against the control sample β-actin (housekeeping protein, anti- β-actin antibodies, Thermofisher, MA, USA, Cat # MA5-15739) by protein normalization.

### X-ray examination of knee joint morphometry

At the end of the experiment, rats were anesthetized, and injected knee joints were examined using a digital vet X-ray (Poskom Co., Ltd., Korea). Structural changes of the tibia and the femoral area between the epiphysial growth plates were evaluated, as well as the articular cartilage. Images were evaluated by two special radiologists who were blinded to the experiment, each radiologist observed the images twice, and the mean scores of both observers were calculated. The Kellgren-Lawrence grading scale was used. The scale ranges from 0 to 4. A score of 0 indicates that there is no evidence of osteoarthritis; a score of 1 indicates the possibility of joint space narrowing and osteophyte formation; a score of 2 indicates definite osteophyte formation and possible joint space narrowing; a score of 3 indicates multiple osteophytes, definite joint space narrowing, sclerosis, and possibly bone deformity; a score of 4 indicates end-stage [50].

### Histopathological examination

From each rat, bones at the mid femur and mid tibia were cut to include both femoral condyles, tibial plateau and whole knee joint in examined histopathologic biopsies. The soft tissue around the bones was dissected. The joints were then fixed in 10% buffered formalin for one day. Decalcification was done by immersion of joints in EDTA for one week. The EDTA solution was changed every 3 days. The decalcified joints were sectioned at the anteroposterior plane (at the level of the patella) and placed in cassettes with their medial side facing the bottom of the cassettes. They were then dehydrated in ascending degrees of alcohol and cleared in xylene before embedding in paraffin. The paraffin blocks were sectioned using a rotatory microtome. Hematoxylin and eosin (H&E), as well as safranin O fast green stained sections (SOC-IFU, ScyTek Laboratories, Inc., USA), were prepared according to the manufacturer’s data sheets. A microscope-adopted digital camera (Leica, EC4 digital microscope camera) was used to capture images of the infrapatellar fat pad (IPF) and the surface of the patellofemoral joint. Histomorphic analysis of images was done by Leica application suite version 4.12 software to assess articular cartilage thickness in microns in the patellofemoral joint. It was measured on the femoral side within the central part according to Takahashi et al. [48]. The degenerative changes of osteoarthritis were then graded by the OARSI cartilage histopathology assessment system [38]. The IPF synovitis and fibrosis were scored (0–3 for each parameter, with a final summed score out of 6) according to M. Udo et al. [52]. Two representative serial sections of each joint were examined and the mean score of the joint was then calculated. All assessments were done in a blind manner by two pathologists who observed the slides twice and the mean scores were calculated.

### Statistical analysis

Values were expressed as means ± SEM (*n* = 10). One-way analysis of variance (ANOVA) was used for statistical analysis of the results, followed by the Tukey Kramer test as the post hoc test. For radiology, knee bend test, and histopathology scores, nonparametric data analysis using the Kruskal–Wallis Test followed by Dunn's post hoc test was performed. Results were presented as median with range. *P* < 0.05 was considered as the significance limit for all comparisons. For the radiology and histopathology examination, the Kappa (κ) test for agreement was used. The intra-class correlation coefficient (ICC) was also used for the agreement between each two observers. The ICCs were classified using a system suggested by McGraw and Wong11 as follows: less than 0.75 Z poor agreement; 0.75 to less than 0.90 Z moderate agreement; 0.90 or greater Z high agreement. The data were analyzed, and graphs were drawn using the GraphPad Prism 5.0 software (GraphPad Software Inc., CA, USA).

## Results

### Effect of diminazene (DIZE) and losartan (LOS) on osteoarthritis-associated behaviors

#### Effect of DIZE and LOS on motor function using the rotarod test

**Fig. 1:**
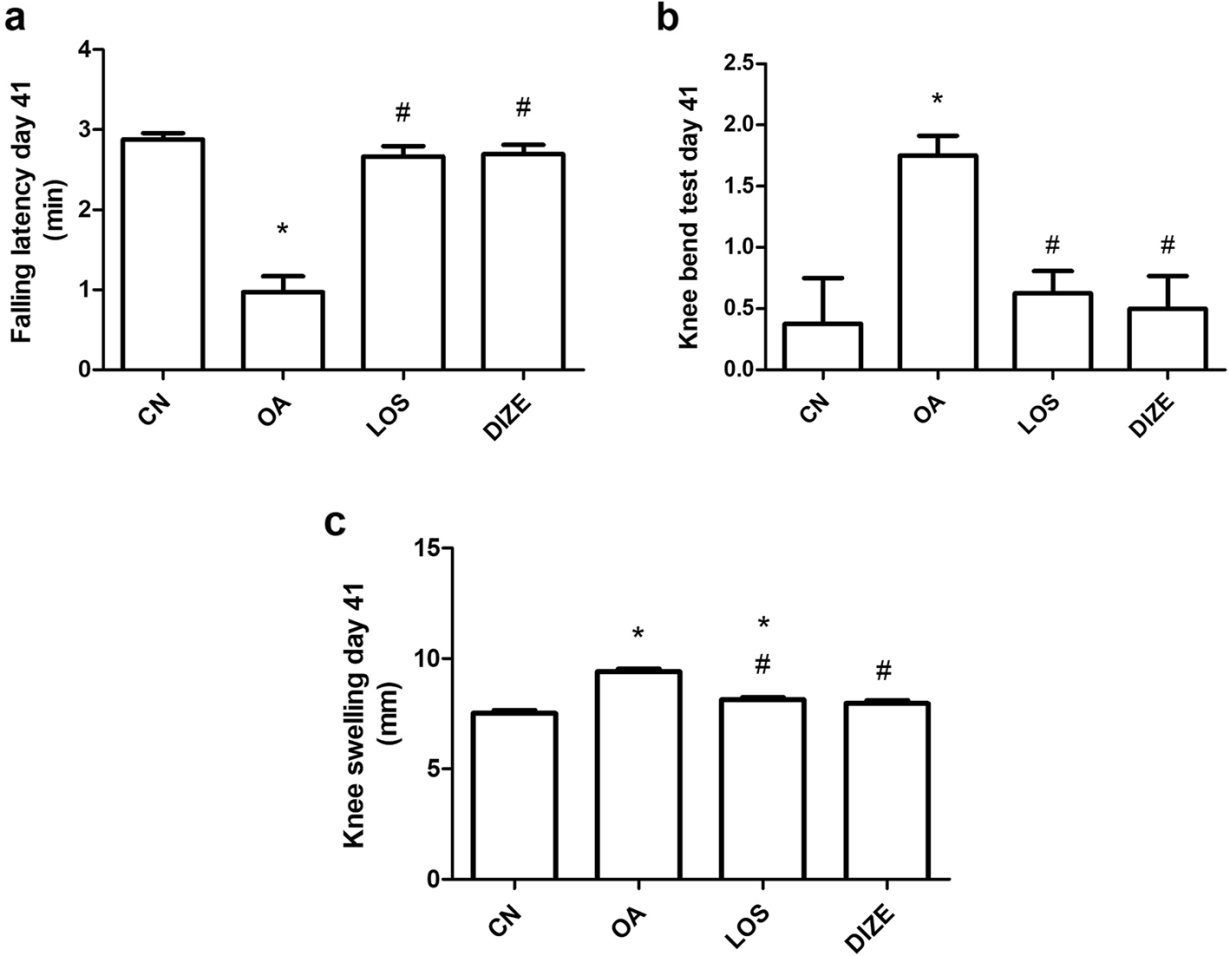
Evaluation of nociception using rotarod (**a**), knee bend (**b**) and knee swelling (**c**) tests. Drugs were administered daily for 21 days. Data are presented as means ± SEM (*n* = 10). Comparisons among groups were analyzed using one-way ANOVA followed by Tukey post-hoc test and comparisons between groups in knee bend test were analyzed using Kruskal–Wallis test, followed by Dunn's post hoc-test. Data are compared at *p* < *0.05* with CN (*), OA (#). CN; control, OA; MIA induced osteoarthritis, LOS; losartan treated osteoarthritis and DIZE; diminazene treated osteoarthritis

The changes in rotarod measurements caused by OA in treated or untreated adult male rats are shown in Fig. 1a. Compared with the respective values of the negative control group, the administration of MIA at a dose of 3mg/kg intraarticularly resulted in a significant decrement in rotarod measurements on day 41 of the study, showing ~ 60% decline in performance compared to the control rats.

The daily administration of both DIZE (15 mg/kg/day) and LOS (20 mg/kg/day) caused significant equipotent improvement in rotarod measurements compared to the untreated OA group (66.19% of change for OA vs11.13% for LOS and 10.30% for DIZE). The figure showing the % of change of rotarod performance after treatment (day 41) compared to day zero is shown in Additional file 2 to this manuscript.

#### Effect of DIZE and LOS on OA-associated knee pain using knee bent test

On day 20 before treatment, all OA rats showed a significant and dramatic increase in signs of pain and vocalization to maximal flexion or extension (mean score 1.750, Table 1). These effects were significantly reversed in both LOS and DIZE-treated groups on day 41 (mean score 0.63 LOS, 0.50 DIZE), showing ~ 44% reduction for LOS and ~ 67% reduction for DIZE (Fig. 1b).

**Table 1:** The knee bend test before and after treatment

	Day 20	Day 41
CN	0.000 ± 0.000	0.375 ± 0.263
OA	1.250^*^ ± 0.250	1.750^*^ ± 0.164
LOS	1.125^*^ ± 0.227	0.625^#^ ± 0.183
DIZE	1.500^*^ ± 0.183	0.500^#^ ± 0.267

Comparisons among groups were analyzed using Kruskal–Wallis Test followed by Dunn's post hoc test. Data are compared at *p* < 0.05 with CN (*), OA (#). Values are presented as means ± S.E.M (*n* = 10)

*CN* control, *OA* MIA induced osteoarthritis, *LOS* losartan treated osteoarthritis and *DIZE* diminazene treated osteoarthritis

### Effect of DIZE and LOS on knee swelling using a vernier caliper

Results showed that OA induction paralleled with a significant increase in knee circumference on day 41 compared to the control rats revealing signs of inflammation **(**Fig. 1c**)**. Treatment with either LOS or DIZE caused a significant reversal of the knee diameter increase compared to OA rats (*p* < 0.0001 for LOS and *p* < 0.0001 for DIZE).

### Effect of DIZE and LOS on osteoarthritic X-ray imaging

**Fig. 2:**
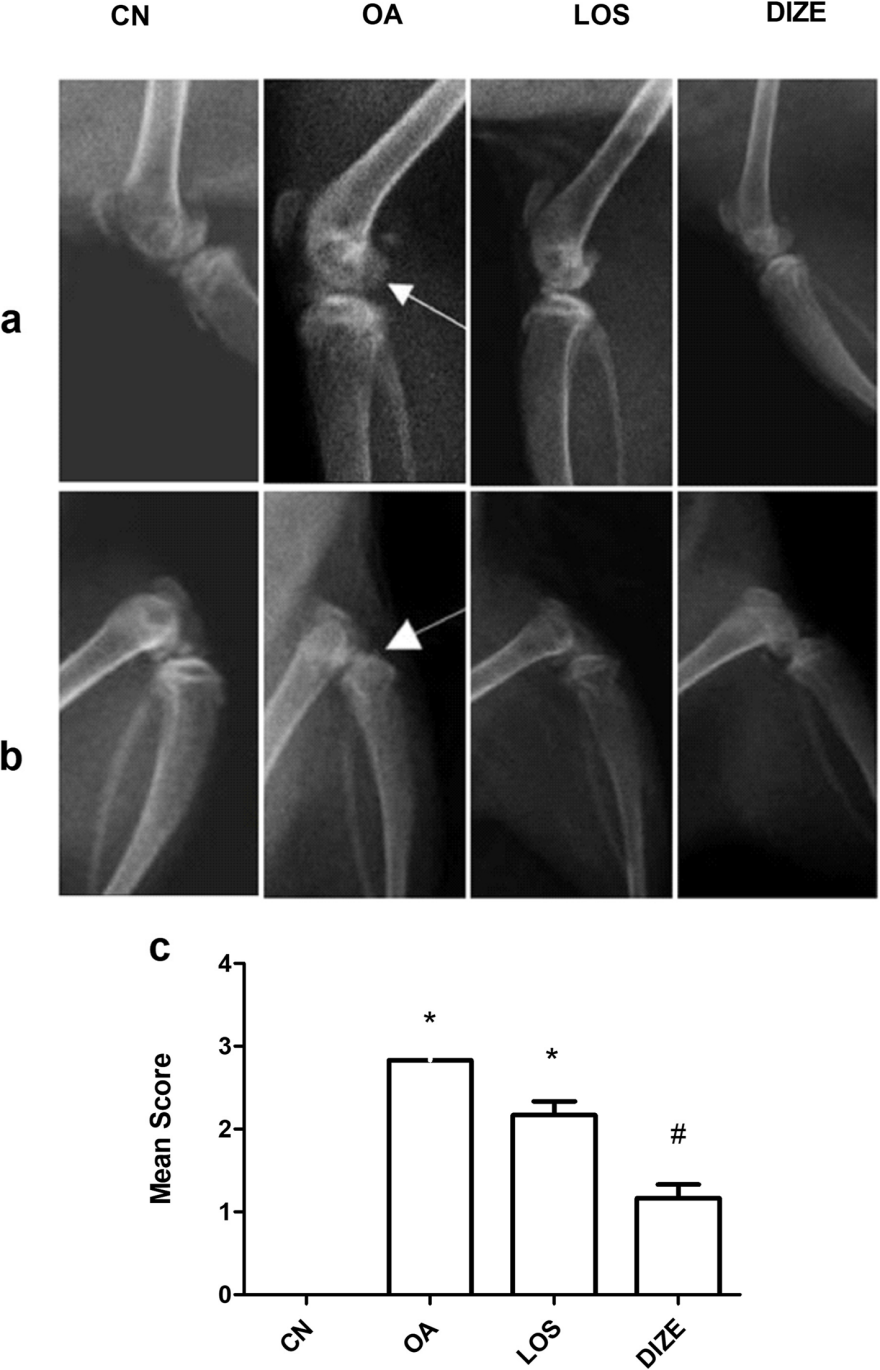
Evaluation of drug treatment on radiological changes of the right knee joint. Panel (**a**) shows representative ventrodorsal view radiographs and panel (**b**) shows representative lateral view radiographs of CN; control, OA; MIA induced osteoarthritis, LOS; losartan treated osteoarthritis and DIZE; diminazene treated osteoarthritis. **c** The mean radiographic score. Drugs were administered daily for 21 days. Data are presented as means ± SEM (*n* = 4). Comparisons among groups were analyzed using one-way ANOVA followed by Tukey post-hoc test. Data are compared at *p* < *0.05* with CN (*) and OA (#)

Radiographic examination of OA rats revealed significant narrowing in the joint space compared to normal rats (Fig. 2a-b). Articular margins have been shown to be sclerotic, and rough with abundant osteophytes in both ventrodorsal and lateral views of OA rats. DIZE treatment shows a better effect than LOS, showing a decrease in the joint space narrowing, and the surface appears smooth with a significant decrease in osteophytes number. The result of the mean score is shown in Fig. 2c. The kappa coefficient of agreement has shown a good reliability of > 0.70 **(**Table 2**)**, while the ICC has shown a good level of agreement between both observers of > 0.80 (Table 3).

**Table 2:** Kappa test for radiology and histopathology

κ (p)	Inter-rater	Intra-rater
OARSI grade (0–6)	0.690 (0.015^*^)	0.885(0.013^*^)
Synovitis (0–3)	0.659 (0.023^*^)	0.523 (0.293)
Fibrosis (0–3)	0.704 (0.084)	0.704 (0.084)
Total IPF	0.779 (0.018^*^)	0.696 (0.020^*^)
Radiographic score	0.736 (0.027^*^)	0.800 (0.003^*^)

*κ *kappa test

^*^Statistically significant at *p* ≤ 0.05

**Table 3:** Intra-class correlation coefficient for radiology and histopathology

	**ICC coefficient**	**95% C.I**	***p***	**ICC coefficient**	**95% C.I**	***p***
Articular cartilage thickness (microns)	0.884	0.701 – 0.958	< 0.001^*^	0.793	0.923 – 0.990	< 0.001^*^
OARSI grade (0–6)	0.927	0.766 – 0.978	< 0.001^*^	0.966	0.885 – 0.990	< 0.001^*^
Synovitis (0–3)	0.830	0.512 – 0.948	< 0.001^*^	0.761	0.359 – 0.925	0.001^*^
Fibrosis (0–3)	0.837	0.528 – 0.950	< 0.001^*^	0.837	0.528 – 0.950	< 0.001^*^
Total IPF	0.950	0.835 – 0.985	< 0.001^*^	0.893	0.671 – 0.968	< 0.001^*^
Radiographic score	0.877	0.629 – 0.963	< 0.001^*^	0.836	0.526 – 0.950	< 0.001^*^

*CI* Confidence interval

^*^Statistically significant at *p* ≤ 0.05

### Effect of DIZE and LOS on the biochemical parameters

**Fig. 3:**
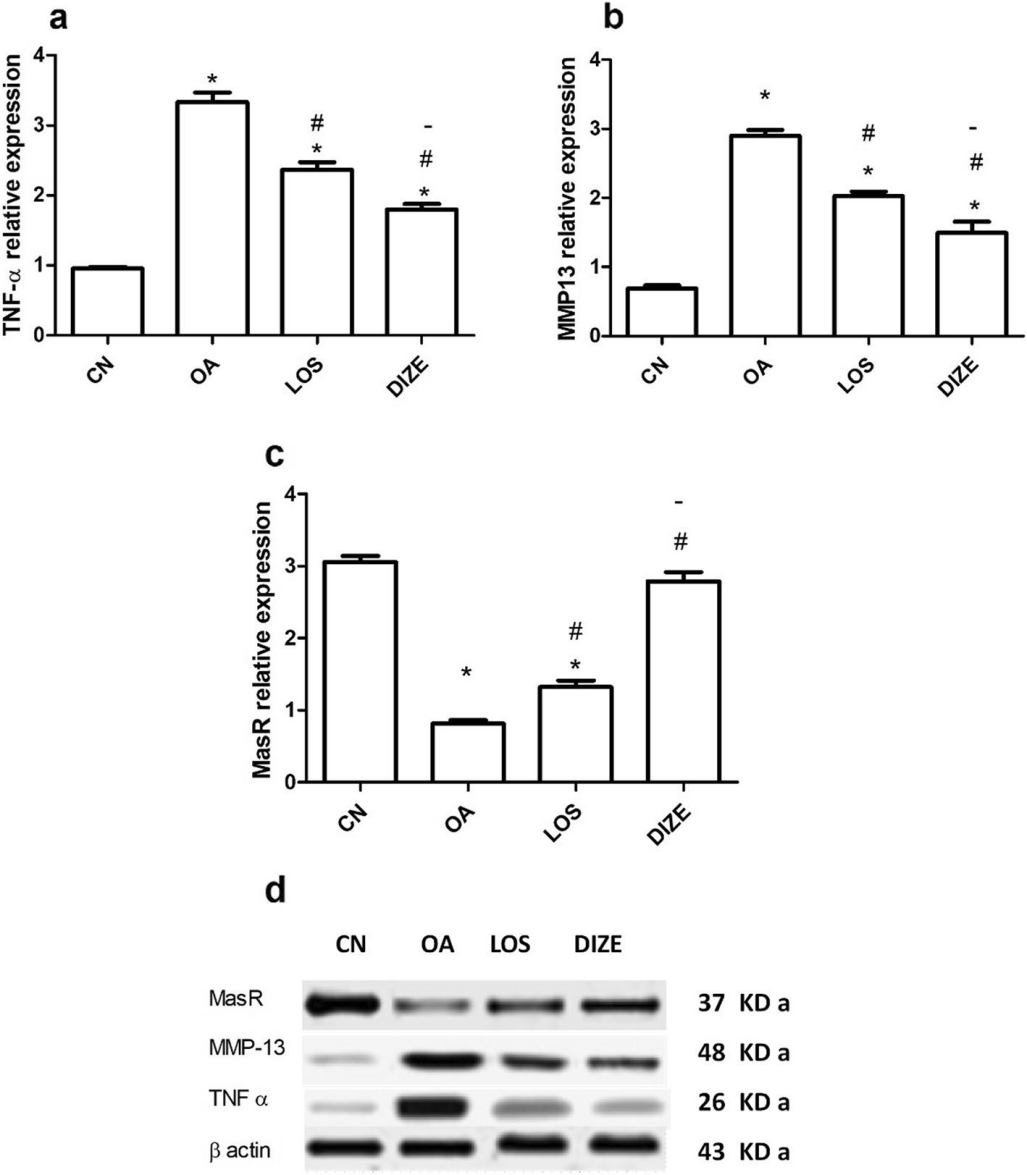
Effect of individual drug treatments on western blot parameters. Tissue levels of (**a**) tumor necrosis factor-alpha (TNF-α), (**b**) matrix metalloproteinases 13 (MMP13) and (**c**) mas receptor (MasR). The western blot bands are grouped from different gels and presented in (**d**). Drugs were administered daily for 21 days. Data are presented as means ± SEM (*n* = 3). Comparisons among groups were analyzed using one-way ANOVA followed by Tukey post-hoc test. Data are compared at *p* < *0.05* with CN (*), OA (#) and LOS (-). CN; control, OA; MIA induced osteoarthritis, LOS; losartan treated osteoarthritis and DIZE; diminazene treated osteoarthritis

Tissue levels of TNF-α, MMP-13 and MasR, markers of inflammation, oxidative stress and RAAS, respectively, were measured by Western technique. Compared with adult healthy control rats, knee tissue levels of TNF-α (Fig. 3a) and MMP-13 (Fig. 3b) were significantly elevated in OA rats, while the MasR (Fig. 3c) decreased. The altered levels in these RAAS, inflammatory and oxidative signals were significantly restored in all treated rats. Among the two drug therapies, the antitrypanosomal drug DIZE showed a more potent effect than the ARB drug LOS (*p* < 0.001 TNF-α, *p* < 0.0001 MasR,* p* < 0.05 MMP-13). Figure 3d shows the grouping of the western blot bands from different fields.

**Fig. 4:**
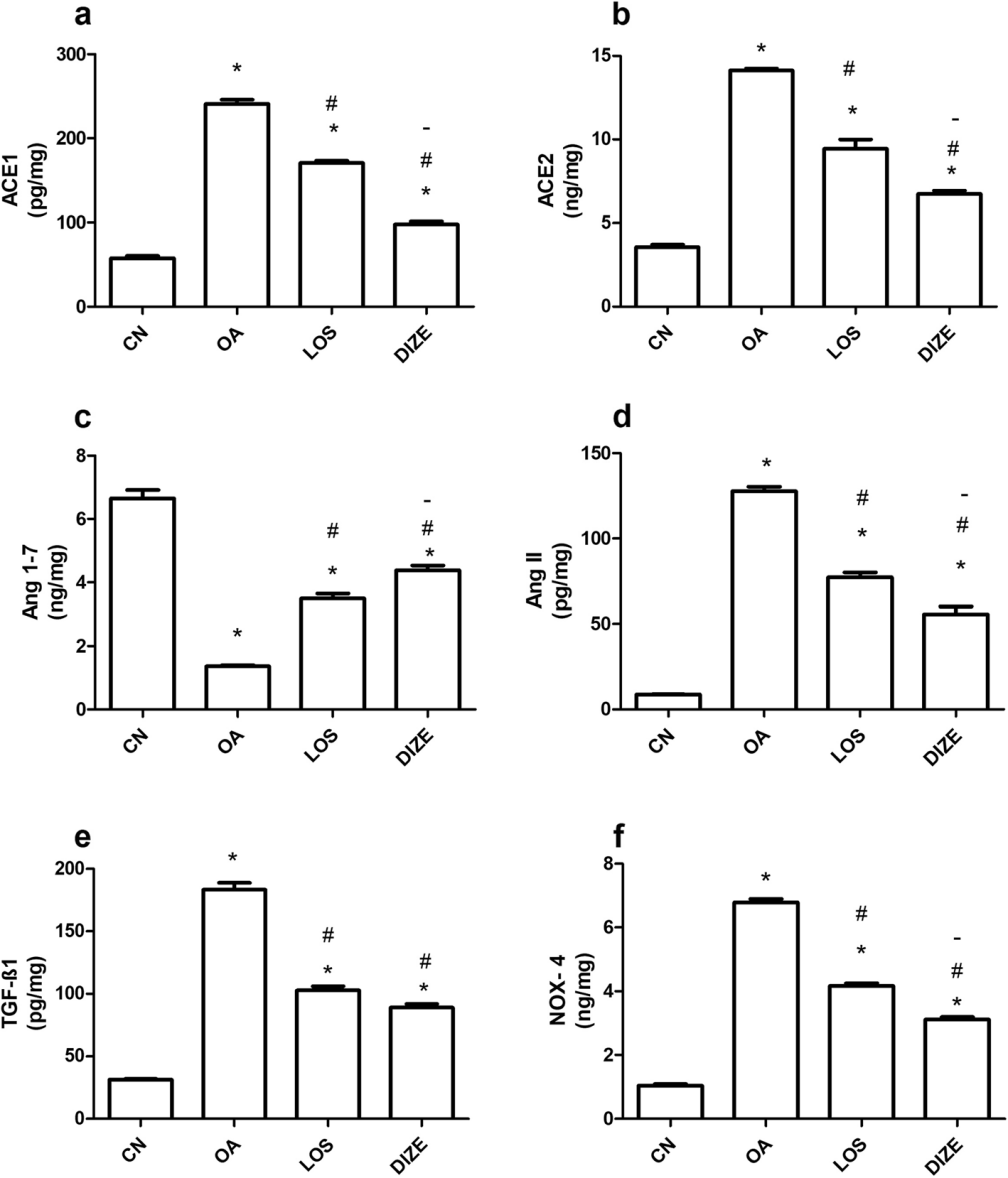
Effect of individual drug treatments on the biochemical parameters. Tissue levels of (**a**) angiotensin-converting enzyme 1 (ACE1), (**b**) angiotensin-converting enzyme 2 (ACE2), (**c**) angiotensin (Ang) 1–7, (**d**) Ang II, (**e**) transforming growth factor beta 1 (TGF-β1) and (**f**) NADPH oxidases 4 (NOX-4). Drugs were administered daily for 21 days. Data are presented as means ± SEM (*n* = 6). Comparisons among groups were analyzed using one-way ANOVA followed by Tukey post-hoc test. Data are compared at *p* < *0.05* with CN (*), OA (#) and LOS (-). CN; control, OA; MIA induced osteoarthritis, LOS; losartan treated osteoarthritis and DIZE; diminazene treated osteoarthritis

ELISA measurements showed that compared with normal healthy knee tissues, OA induction resulted in a significant elevation of ACE1, ACE2, Ang II, TGF-β1 and NOX-4 levels in knee tissues (Fig. 4), while the Ang 1–7 level was significantly reduced (Fig. 4c). Such effects were reversed upon treatment with LOS or DIZE, where DIZE effectively caused a more significant change than LOS in almost all studied parameters (*p* < 0.0001 ACE1, *p* < 0.0001 ACE2, *p* < 0.001 Ang 1–7, *p* < 0.0001 Ang II, *p* < 0.0001 NOX-4), except TGF-β1, where both showed to be equally effective (Fig. 4e).

### Effect of DIZE and LOS on knee histopathology

**Fig. 5:**
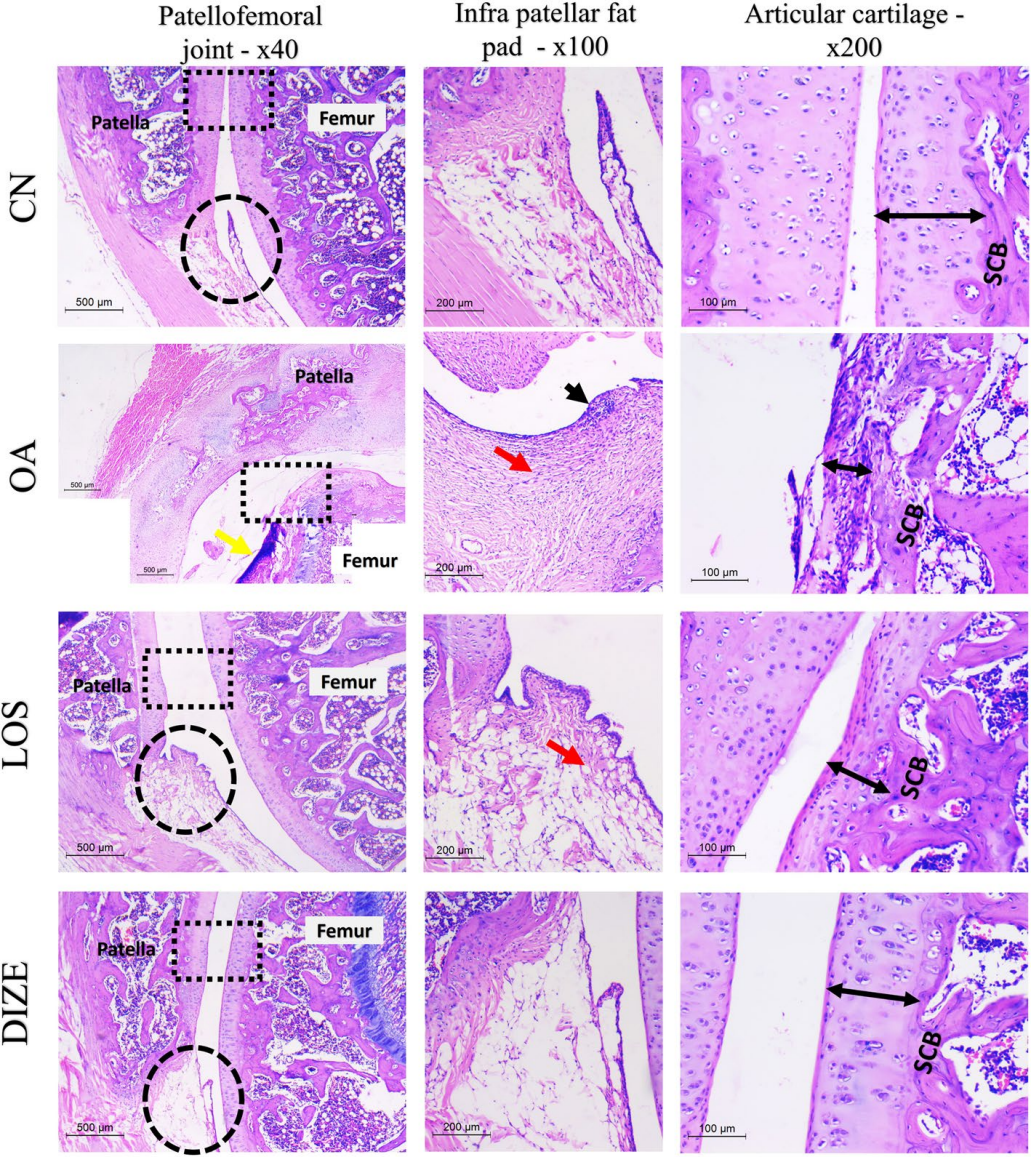
H&E stained sections of the patellofemoral joint in studied groups. The first column represents low power × 40 (scale bar = 500 microns). The second column shows the high power of the round dashed area to represent the infrapatellar fat pad (IPF) changes (× 100, scalebar = 200 microns). The third column is the high power of the squared dashed area to show the articular cartilage thickness (double-headed arrow) and subchondral bone (SCB) (× 200, scalebar = 100 microns). The control (CN) group shows a normal architecture of the joint, IPF is formed of fat with no synovitis or fibrosis, and articular cartilage is thick with preserved zones. The subchondral bone is thin. The OA group shows joint deformities of articular cartilage (yellow arrow). The IPF shows inflammation (black arrow) with severe fibrosis (red arrow) and no fat cells are seen (score 6). Articular cartilage is thinned out and composed of a fibrous cap. SCB is thick and sclerosed. The changes were improved in the LOS and DIZE groups. The LOS group shows improved inflammation in IPF with residual fibrosis (black arrow). High power shows increased thickness of cartilage with residual mild surface irregularities and thin SCB. Meanwhile, the DIZE group showed smooth cartilaginous surfaces of the patellofemoral joint and resolved inflammation and fibrosis in IPF. Articular cartilage showed organized chondrocytes with no clustering or proliferation, and thin SCB. *Yellow arrow* = *joint deformities, SCB* = *subchondral bone, black arrow* = *inflammation, red arrow* = *fibrosis, double head arrow* = *articular cartilage thickness*

**Fig. 6:**
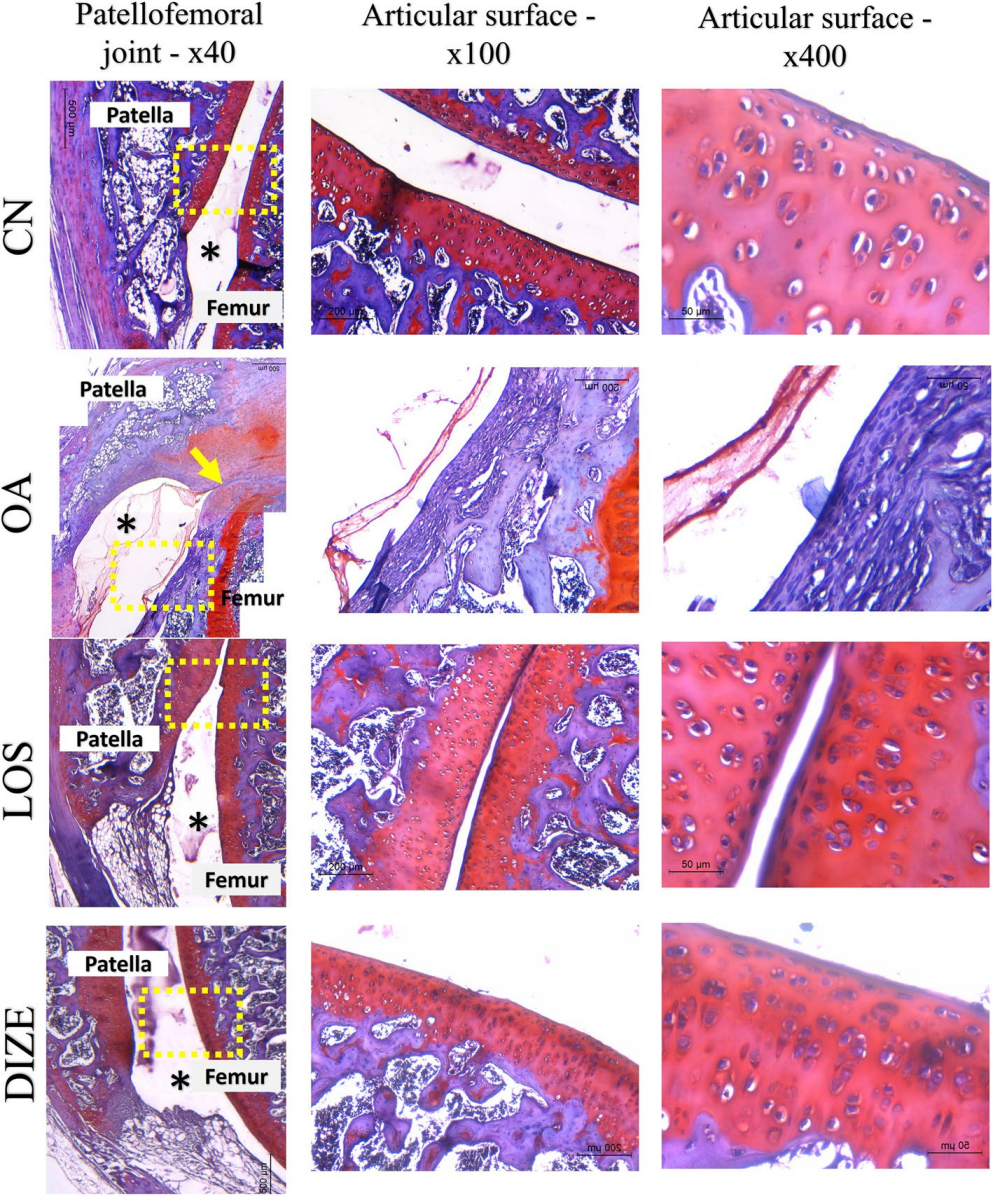
Safranin O fast green stained sections of the patellofemoral joint in studied groups. The first column represents low power × 40. The second column shows higher power of square dashed area to the articular cartilage (× 100, scale bar = 200 microns). The third column is a higher power to show the articular cartilage proteoglycan content (× 400, scalebar = 50 microns). The control (CN) group shows a normal architecture of the joint with preserved joint space (*). Higher power reveals a smooth cartilaginous surface with a deep red color indicating high PG content. The highest power shows organized chondrocytes. The OA group shows joint space narrowing (yellow arrow) with severe joint effusion (*). High power shows a total loss of cartilaginous surface. The highest power shows fibrous cap replacing cartilage. The changes were improved in the LOS and DIZE groups. Both groups show restoration of joint space (*) with widening indicating mild effusion. The articular surfaces are smooth with only fine mild irregularities. High power shows viable chondrocytes in deep red matric (increased PG content)

**Fig. 7:**
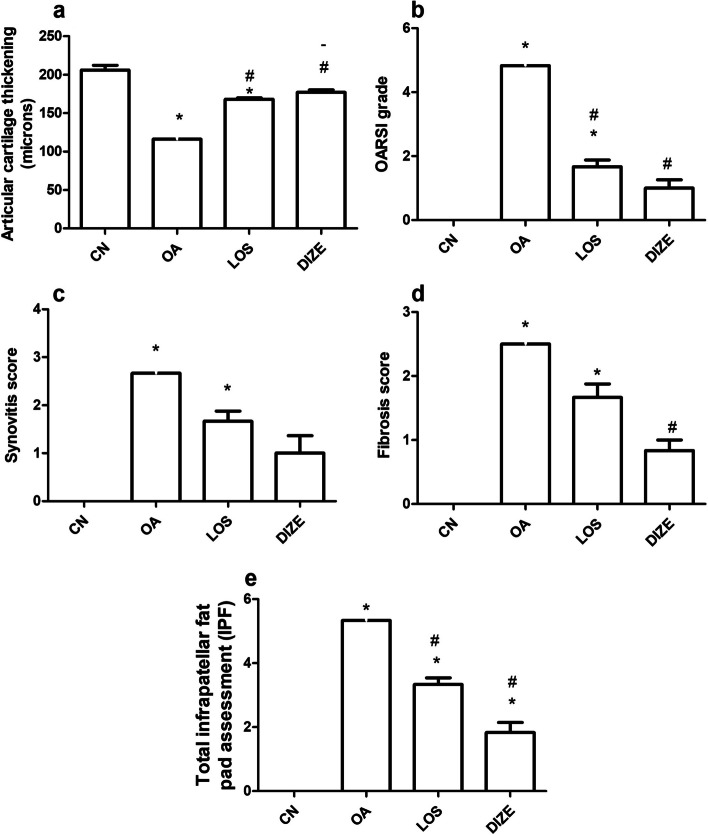
Effect of individual drug treatments on the histomorphic changes of the patellofemoral joint in H&E and safranin O fast green stained sections. **a** Articular cartilage thickening, **b** OARSI grade, **c** synovitis, **d** fibrosis and **e** total infrapatellar fat pad (IPF). The total IPF assessment resembles the total score of synovitis and fibrosis. Data are presented as means ± SEM (*n* = 4). Comparisons among groups were analyzed using one-way ANOVA followed by Tukey post-hoc test for the articular cartilage thickening assessment. Kruskal–Wallis test, followed by Dunn's post-hoc test was used to analyze the OARSI grade and IPF scores. Data are compared at *p* < 0.05 with CN (*), OA (#), and LOS (-) at *p* < *0.05*. CN; control, OA; MIA induced osteoarthritis, LOS; losartan treated osteoarthritis and DIZE; diminazene treated osteoarthritis

Histomorphic changes of the patellofemoral joint in H&E and safranin O fast green stained sections are shown in Figs. 5, 6, and 7. The CN groups showed normal architecture of patellofemoral joints with preserved joint space. The total infrapatellar fat pad assessment (IPF) was formed of adipose tissue with no synovitis or fibrosis. The patella and femur were covered by a regular layer of articular cartilage (202.1 microns). It is formed of thick hyaline cartilage overlying a thinner layer of calcified cartilage. The hyaline cartilage showed a smooth surface and was composed of viable properly oriented chondrocytes. They were arranged in three layers (superficial, mid, and deep). No clustering or proliferation is present. The subchondral bone plate was thin. Both OARSI grade and IPF score were zero (Fig. 7b and e). Osteoarthritis-related degenerative changes were evident in the OA group with deformed joints and narrow joint space. The articular cartilage was thinned out (122.3 microns). The ratio of calcified to hyalinized cartilage was increased with evident thickening of subchondral bone. At the same time, IFP showed evident synovitis. The latter was seen as thickened hyperplastic synovium with subsynovial inflammatory infiltrate. Severe fibrosis was noted with total loss of fat cells in some areas (IPF score 4–6). The articular cartilage showed denuded areas and was replaced by fibrous cap in other areas. (OARSI grade 4–6) Markedly diminished PG content is seen in safranin O fast green stain. Both LOS and DIZE-studied groups were able to reverse the degenerative changes of osteoarthritis. In LOS-treated rats, the OARSI grade dropped to a score (1–2), the IPF score was (3–4), and synovitis was much improved, however, fibrosis was still seen in some rats. The articular cartilage thickness was shown to be 167.5 microns. Meanwhile, the DIZE group showed a better improvement. The articular cartilage restored its thickness to be 178.6 microns. The OARSI grade was (1–2), synovitis, and fibrosis were minimal of IPF score 0–1. The articular cartilage regained its normal architecture with the preservation of the three zones of hyaline cartilage. Degenerated chondrocytes were only occasionally seen. The kappa coefficient of agreement has shown a good reliability of > 0.60 (Table 2), while the ICC has shown a good to excellent level of agreement between both observers of > 0.80 and > 0.90 (Table 3).

## Discussion

The present study examined the effects of the ACE2 activator, diminazene aceturate to tone down joint anomalies in an osteoarthritis rat model, in comparison with the indirect ACE2 activator, losartan, a frequently used cardiovascular drug. Induction of knee osteoarthritis caused a loss in muscle coordination, knee swelling, elevations in the inflammatory mediators (TNF-α & TGF-β1), and the oxidative stress markers (MMP-13 & NOX-4) in joint tissues. Furthermore, knee joint tissues of OA rats showed increased levels of Ang II, ACE1, ACE2, decreased Ang 1–7 and MasR expression. Both ACE2 activator drugs, diminazene and losartan reversed all OA behavioral, radiological and histopathological anomalies, together with a decline in knee inflammation and oxidative stress markers. These beneficial changes in the knee occurred in parallel with a reduction of the harmful RAAS arm (ACE1-AngII) and an enhancement of the beneficial RAAS arm (ACE2-MasR).

Osteoarthritis was induced in the experimental rat model through a single intra-​articular injection of monosodium iodoacetate (MIA) which caused a reduction in the number of chondrocytes, besides causing many histological and radiographic changes in both the articular cartilage and synovial membrane. Histopathological examination in the current study came to confirm the development of OA showing thinning in the articular cartilage, in addition to synovitis and fibrosis in knee joints of OA untreated rats as stated in earlier studies [11, 19].

In rheumatoid arthritis and OA knee animal models, cartilage injury results in abnormal motor activity and coordination [35, 42], which is reflected in the rotarod performance of OA untreated rats in the current study. Similarly, Ruan et al. showed a decreased time on the rotarod for surgically induced OA in mice [42]. The improvement of motor performance by both diminazene and losartan in the current study might be explained by their proven ability to reduce both oxidative stress and inflammatory parameters [16, 23, 28, 57]. Both ACE2 activator drugs decreased osteoarthritic anomalies, reflected in this study as decreased osteoarthritic knee swelling and improved oxidative and inflammatory parameters.

Knee effusion is a common symptom among people suffering from osteoarthritic knee, characterized by knee pain and swelling [24]. The visible inspection of knee swelling, and the knee bend test is then considered reliable parameters for the measurement of improved osteoarthritic joint effusion in animal models of OA [8, 10, 61]. Diminazene and losartan were able to significantly decrease the knee swelling and pain observed in the current OA model. The effect of losartan on osteoarthritic knee effusion has been noticed previously by Thomas et al., where losartan was able to attenuate the progression of OA in knee joints of a chondrodysplasia mouse model, reducing the degeneration of knee joint articular cartilage and halting the inflammatory process through the inhibition of TGF-β1 signaling pathway [51]. This was further confirmed by the study of Abdel El-Gaphar et al. which showed a decreased rat arthritic inflammation by losartan via decreasing inflammatory mediators and oxidative stress markers [1]. Similarly, TGF-β1 level has shown a significant decrease upon treatment with losartan or diminazene compared to OA rats in this study.

The RAAS is found normally in the body and it is essential for regulating normal body homeostasis and balance, besides its major role in controlling inflammation [27]. Many studies revealed that RAAS-related components have a major role in the etiology of OA and rheumatoid arthritis [26, 45, 56]. A review article has shown that both RAAS axes, the classical one, which is formed from ACE, Ang II and AT-1R, and the counter-regulatory axes, composed by ACE2, Ang 1–7 and the MasR, modulate inflammation and tissue damage in rheumatoid arthritis disease. While Ang II activates pro-inflammatory mediators and oxidative stress molecules, Ang 1–7 exerts anti-inflammatory actions by decreasing cytokine release (ref missing). Khajeh Pour S. et al. examined the anti-inflammatory effect of Ang 1–7 on an adjuvant-induced arthritis rat model, demonstrating its positive effect on the swelled joint causing a significant decrease in nitric oxide (NO) levels when compared with inflamed animals [18]. Moreover, after administration of Ang 1–7 there was a reduction in the mRNA expression of ACE1, ACE2, AT-1R, and MasR in different tissues when compared with the diseased model. Hereby, the progression of OA in the current study caused a significant increase in ACE1, ACE2, Ang II and a decrease in Ang 1–7 and MasR. It is noteworthy that diminazene treatment showed better control of the RAAS-related inflammatory components than losartan.

The NOX-4 isoform is expressed in chondrocytes and has shown involvement in the production of catabolic proteases such as MMP-13 molecules, which in turn increase oxidative stress in the osteoarthritic cartilage [41]. In line with this data, OA rats in the current study have shown increased levels of TNF-α, TGF-β1, MMP-13 and NOX-4, which were amended upon treatment with losartan or diminazene. Results in the current study came in accordance with the study of Rajapaksha IG et al. that investigated the effect of diminazene aceturate on a mouse model of liver injury and biliary fibrosis, showing that mice treated with diminazene had a significant reduction in both inflammatory and oxidative stress molecules presented as downregulation in the expression of TGF-β1, TNF-α and NOX [39].

Results of the histopathology examination came to confirm the biochemical OA biomarkers” analysis, where treatment with diminazene was able to revert the OA anomalies, observed as restored articular cartilage thickness showing normal architecture, a diminished number of degenerated chondrocytes, minimal synovitis and fibrosis compared to losartan effect.

## Conclusion

In conclusion, the present data highlight the important role of both diminazene aceturate and losartan in ameliorating OA knee dysfunction. However, the direct ACE2 activator drug, diminazene, has shown a more prominent effect in almost all behavioral, radiological, biological and histopathological anomalies. The role of the RAAS signaling pathway, especially the ACE2/Ang1-7/MasR axis, proved its role in the immunomodulatory and anti-inflammatory effect in OA. To our knowledge, this is the first study to explore the effect of direct ACE2 activator diminazene in the osteoarthritic model and nominate ACE2/Ang1-7/MasR cascade as a potential molecular target for disease-modifying anti-osteoarthritic drugs.

### Supplementary Information

**Additional file 1.** Shows the principle and procedure of the ELISA assay.

**Additional file 2.** Shows the % of change of rotarod performance.

**Additional file 3.** Shows the original western blot bands.

## Data Availability

All data generated or analysed during this study are included in this published article.

## Abbreviations

ACE2: Angiotensin-converting enzyme
Ang: Angiotensin
ARBs: Angiotensin receptor blockers
DIZE: Diminazene
ECM: Extracellular matrix
LOS: Losartan
NOX-4: NADPH oxidase-4
OA: Osteoarthritis
RAAS: Renin–angiotensin–aldosterone system
ROS: Reactive oxygen species

## References

[1] Abdel El-Gaphar OAM, Abo-Youssef AM, Abo-Saif AA (2018). Effect of losartan in complete Freund's adjuvant -induced arthritis in rats. Iran J Pharm Res.

[2] Abuohashish HM, Ahmed MM, Sabry D, Khattab MM, Al-Rejaie SS (2017). ACE-2/Ang1-7/Mas cascade mediates ACE inhibitor, captopril, protective effects in estrogen-deficient osteoporotic rats. Biomed Pharmacother.

[3] Abuohashish HM, Ahmed MM, Sabry D, Khattab MM, Al-Rejaie SS (2017). Angiotensin (1–7) ameliorates the structural and biochemical alterations of ovariectomy-induced osteoporosis in rats via activation of ACE-2/Mas receptor axis. Sci Rep.

[4] Akhtar S, Benter IF, Danjuma MI, Doi SAR, Hasan SS, Habib AM (2020). Pharmacotherapy in COVID-19 patients: a review of ACE2-raising drugs and their clinical safety. J Drug Target.

[5] Awwad ZM, El-Ganainy SO, ElMallah AI, Khedr SM, Khattab MM, El-Khatib AS (2020). Assessment of pregabalin-induced cardiotoxicity in rats: mechanistic role of angiotensin 1–7. Cardiovasc Toxicol.

[6] Cobankara V, Oztürk MA, Kiraz S, Ertenli I, Haznedaroglu IC, Pay S, Calgüneri M (2005). Renin and angiotensin-converting enzyme (ACE) as active components of the local synovial renin-angiotensin system in rheumatoid arthritis. Rheumatol Int.

[7] Egloff C, Hügle T, Valderrabano V (2012). Biomechanics and pathomechanisms of osteoarthritis. Swiss Med Wkly.

[8] Ferreira-Gomes J, Adães S, Castro-Lopes JM (2008). Assessment of movement-evoked pain in osteoarthritis by the knee-bend and CatWalk tests: a clinically relevant study. J Pain.

[9] Fortuño A, Bidegain J, Robador PA, Hermida J, López-Sagaseta J, Beloqui O, Díez J, Zalba G (2009). Losartan metabolite EXP3179 blocks NADPH oxidase-mediated superoxide production by inhibiting protein kinase C: potential clinical implications in hypertension. Hypertension.

[10] Foureaux G, Nogueira BS, Coutinho DC, Raizada MK, Nogueira JC, Ferreira AJ (2015). Activation of endogenous angiotensin converting enzyme 2 prevents early injuries induced by hyperglycemia in rat retina. Braz J Med Biol Res.

[11] Goldring MB, Goldring SR (2007). Osteoarthritis. J Cell Physiol.

[12] Griendling KK, Minieri CA, Ollerenshaw JD, Alexander RW (1994). Angiotensin II stimulates NADH and NADPH oxidase activity in cultured vascular smooth muscle cells. Circ Res.

[13] Hagiwara H, Hiruma Y, Inoue A, Yamaguchi A, Hirose S (1998). Deceleration by angiotensin II of the differentiation and bone formation of rat calvarial osteoblastic cells. J Endocrinol.

[14] Hanafy AS, El-Ganainy SO (2020). Thermoresponsive Hyalomer intra-articular hydrogels improve monoiodoacetate-induced osteoarthritis in rats. Int J Pharm.

[15] Hatton R, Stimpel M, Chambers TJ (1997). Angiotensin II is generated from angiotensin I by bone cells and stimulates osteoclastic bone resorption in vitro. J Endocrinol.

[16] Jia F, Zhang X, Ma W, Li X, Zhou X (2021). Cytotoxicity and anti-inflammatory effect of a novel diminazene aceturate derivative in bovine mammary epithelial cells. Res Vet Sci.

[17] Kawakami Y, Matsuo K, Murata M, Yudoh K, Nakamura H, Shimizu H, Beppu M, Inaba Y, Saito T, Kato T, Masuko K (2012). Expression of angiotensin II receptor-1 in human articular chondrocytes. Arthritis.

[18] Khajeh Pour S, Ranjit A, Summerill EL, Aghazadeh-Habashi A (2022) Anti-Inflammatory Effects of Ang-(1–7) Bone-Targeting Conjugate in an Adjuvant-Induced Arthritis Rat Model. Pharmaceuticals (Basel) 15. 10.3390/ph15091157 10.3390/ph15091157 PMC9502795 36145378

[19] Kuyinu EL, Narayanan G, Nair LS, Laurencin CT (2016). Animal models of osteoarthritis: classification, update, and measurement of outcomes. J Orthop Surg Res.

[20] Laferriere CA, Pang DS (2020). Review of intraperitoneal injection of sodium pentobarbital as a method of euthanasia in laboratory rodents. J Am Assoc Lab Anim Sci.

[21] Lei C, Wu S, Wen C, Li Y, Liu N, Huang J, Li L, Fu M, Liu J (2019). Zafirlukast attenuates advanced glycation end-products (AGEs)-induced degradation of articular extracellular matrix (ECM). Int Immunopharmacol.

[22] Liao CR, Wang SN, Zhu SY, Wang YQ, Li ZZ, Liu ZY, Jiang WS, Chen JT, Wu Q (2020). Advanced oxidation protein products increase TNF-α and IL-1β expression in chondrocytes via NADPH oxidase 4 and accelerate cartilage degeneration in osteoarthritis progression. Redox Biol.

[23] Lin CH, Yang H, Xue QL, Chuang YF, Roy CN, Abadir P, Walston JD (2014). Losartan improves measures of activity, inflammation, and oxidative stress in older mice. Exp Gerontol.

[24] Maricar N, Callaghan MJ, Parkes MJ, Felson DT, O'Neill TW (2016). Clinical assessment of effusion in knee osteoarthritis-a systematic review. Semin Arthritis Rheum.

[25] Mei J, Sun J, Wu J, Zheng X (2019). Liraglutide suppresses TNF-α-induced degradation of extracellular matrix in human chondrocytes: a therapeutic implication in osteoarthritis. Am J Transl Res.

[26] Moreira FRC, de Oliveira TA, Ramos NE, Abreu MAD, Simões ESAC (2021). The role of renin angiotensin system in the pathophysiology of rheumatoid arthritis. Mol Biol Rep.

[27] Muñoz-Durango N, Fuentes CA, Castillo AE, González-Gómez LM, Vecchiola A, Fardella CE, Kalergis AM (2016) Role of the Renin-Angiotensin-Aldosterone System beyond Blood Pressure Regulation: Molecular and Cellular Mechanisms Involved in End-Organ Damage during Arterial Hypertension. Int J Mol Sci 17. 10.3390/ijms17070797 10.3390/ijms17070797 PMC4964362 27347925

[28] Nakamura A, Shikata K, Nakatou T, Kitamura T, Kajitani N, Ogawa D, Makino H (2013). Combination therapy with an angiotensin-converting-enzyme inhibitor and an angiotensin II receptor antagonist ameliorates microinflammation and oxidative stress in patients with diabetic nephropathy. J Diabetes Investig.

[29] Neidhart M, Karouzakis E, Jüngel A, Gay RE, Gay S (2014). Inhibition of spermidine/spermine N1-acetyltransferase activity: a new therapeutic concept in rheumatoid arthritis. Arthritis Rheumatol.

[30] Nuki G (1999). Osteoarthritis: a problem of joint failure. Z Rheumatol.

[31] Onozato ML, Tojo A, Goto A, Fujita T, Wilcox CS (2002). Oxidative stress and nitric oxide synthase in rat diabetic nephropathy: effects of ACEI and ARB. Kidney Int.

[32] Onozato ML, Tojo A, Kobayashi N, Goto A, Matsuoka H, Fujita T (2007). Dual blockade of aldosterone and angiotensin II additively suppresses TGF-beta and NADPH oxidase in the hypertensive kidney. Nephrol Dial Transplant.

[33] Patel VB, Zhong JC, Grant MB, Oudit GY (2016). Role of the ACE2/angiotensin 1–7 axis of the renin-angiotensin system in heart failure. Circ Res.

[34] Paul M, Poyan Mehr A, Kreutz R (2006). Physiology of local renin-angiotensin systems. Physiol Rev.

[35] Piel MJ, Kroin JS, van Wijnen AJ, Kc R, Im HJ (2014). Pain assessment in animal models of osteoarthritis. Gene.

[36] Pollard TC, Gwilym SE, Carr AJ (2008). The assessment of early osteoarthritis. J Bone Joint Surg Br.

[37] Porollo A, Meller J, Joshi Y, Jaiswal V, Smulian AG, Cushion MT (2012). Analysis of current antifungal agents and their targets within the Pneumocystis carinii genome. Curr Drug Targets.

[38] Pritzker KP, Gay S, Jimenez SA, Ostergaard K, Pelletier JP, Revell PA, Salter D, van den Berg WB (2006). Osteoarthritis cartilage histopathology: grading and staging. Osteoarthritis Cartilage.

[39] Rajapaksha IG, Mak KY, Huang P, Burrell LM, Angus PW, Herath CB (2018). The small molecule drug diminazene aceturate inhibits liver injury and biliary fibrosis in mice. Sci Rep.

[40] Refaat R, Salama M, Abdel Meguid E, El Sarha A, Gowayed M (2013). Evaluation of the effect of losartan and methotrexate combined therapy in adjuvant-induced arthritis in rats. Eur J Pharmacol.

[41] Rousset F, Hazane-Puch F, Pinosa C, Nguyen MV, Grange L, Soldini A, Rubens-Duval B, Dupuy C, Morel F, Lardy B (2015). IL-1beta mediates MMP secretion and IL-1beta neosynthesis via upregulation of p22(phox) and NOX4 activity in human articular chondrocytes. Osteoarthritis Cartilage.

[42] Ruan MZ, Patel RM, Dawson BC, Jiang MM, Lee BH (2013). Pain, motor and gait assessment of murine osteoarthritis in a cruciate ligament transection model. Osteoarthritis Cartilage.

[43] Santos RA, Simoes e Silva AC, Maric C, Silva DM, Machado RP, de Buhr I, Heringer-Walther S, Pinheiro SV, Lopes MT, Bader M, Mendes EP, Lemos VS, Campagnole-Santos MJ, Schultheiss HP, Speth R, Walther T (2003). Angiotensin-(1–7) is an endogenous ligand for the G protein-coupled receptor Mas. Proc Natl Acad Sci U S A.

[44] Shenoy V, Gjymishka A, Jarajapu YP, Qi Y, Afzal A, Rigatto K, Ferreira AJ, Fraga-Silva RA, Kearns P, Douglas JY, Agarwal D, Mubarak KK, Bradford C, Kennedy WR, Jun JY, Rathinasabapathy A, Bruce E, Gupta D, Cardounel AJ, Mocco J, Patel JM, Francis J, Grant MB, Katovich MJ, Raizada MK (2013). Diminazene attenuates pulmonary hypertension and improves angiogenic progenitor cell functions in experimental models. Am J Respir Crit Care Med.

[45] Shirinsky I, Shirinsky V (2016). SAT0453 does renin-angiotensin-aldosterone system blockade influence pain, function and radiographic progression in knee osteoarthritis? An analysis of osteoarthritis initiative data. Ann Rheum Dis.

[46] Shuai B, Yang YP, Shen L, Zhu R, Xu XJ, Ma C, Lv L, Zhao J, Rong JH (2015). Local renin-angiotensin system is associated with bone mineral density of glucocorticoid-induced osteoporosis patients. Osteoporos Int.

[47] Singh N, Joshi S, Guo L, Baker MB, Li Y, Castellano RK, Raizada MK, Jarajapu YP (2015). ACE2/Ang-(1–7)/Mas axis stimulates vascular repair-relevant functions of CD34+ cells. Am J Physiol Heart Circ Physiol.

[48] Takahashi I, Matsuzaki T, Kuroki H, Hoso M (2018). Induction of osteoarthritis by injecting monosodium iodoacetate into the patellofemoral joint of an experimental rat model. PLoS One.

[49] Tao L, Qiu Y, Fu X, Lin R, Lei C, Wang J, Lei B (2016). Angiotensin-converting enzyme 2 activator diminazene aceturate prevents lipopolysaccharide-induced inflammation by inhibiting MAPK and NF-κB pathways in human retinal pigment epithelium. J Neuroinflammation.

[50] Thomas KA, Kidziński Ł, Halilaj E, Fleming SL, Venkataraman GR, Oei EHG, Gold GE, Delp SL (2020). Automated classification of radiographic knee osteoarthritis severity using deep neural networks. Radiol Artif Intell.

[51] Thomas M, Fronk Z, Gross A, Willmore D, Arango A, Higham C, Nguyen V, Lim H, Kale V, McMillan G, Seegmiller RE (2019). Losartan attenuates progression of osteoarthritis in the synovial temporomandibular and knee joints of a chondrodysplasia mouse model through inhibition of TGF-β1 signaling pathway. Osteoarthritis Cartilage.

[52] Udo M, Muneta T, Tsuji K, Ozeki N, Nakagawa Y, Ohara T, Saito R, Yanagisawa K, Koga H, Sekiya I (2016). Monoiodoacetic acid induces arthritis and synovitis in rats in a dose- and time-dependent manner: proposed model-specific scoring systems. Osteoarthritis Cartilage.

[53] Velkoska E, Patel SK, Griggs K, Pickering RJ, Tikellis C, Burrell LM (2015). Short-term treatment with diminazene aceturate ameliorates the reduction in kidney ACE2 activity in rats with subtotal nephrectomy. PLoS One.

[54] Wang Y, Kou J, Zhang H, Wang C, Li H, Ren Y, Zhang Y (2018). The renin-angiotensin system in the synovium promotes periarticular osteopenia in a rat model of collagen-induced arthritis. Int Immunopharmacol.

[55] Wu Y, Li M, Zeng J, Feng Z, Yang J, Shen B, Zeng Y (2020). Differential expression of renin-angiotensin system-related components in patients with rheumatoid arthritis and osteoarthritis. Am J Med Sci.

[56] Wu Y, Lu X, Li M, Zeng J, Zeng J, Shen B, Zeng Y (2019). Renin-angiotensin system in osteoarthritis: a new potential therapy. Int Immunopharmacol.

[57] Yoon SH, Kang HB, Kim J, Yoo K, Han SJ (2022). Diminazene aceturate attenuates hepatic ischemia/reperfusion injury in mice. Sci Rep.

[58] Zafari AM, Ushio-Fukai M, Minieri CA, Akers M, Lassègue B, Griendling KK (1999). Arachidonic acid metabolites mediate angiotensin II-induced NADH/NADPH oxidase activity and hypertrophy in vascular smooth muscle cells. Antioxid Redox Signal.

[59] Zahan OM, Serban O, Gherman C, Fodor D (2020). The evaluation of oxidative stress in osteoarthritis. Med Pharm Rep.

[60] Zheng C, Lei C, Chen Z, Zheng S, Yang H, Qiu Y, Lei B (2015). Topical administration of diminazene aceturate decreases inflammation in endotoxin-induced uveitis. Mol Vis.

[61] Ziaei A, Sahranavard S, Gharagozlou MJ, Faizi M (2016) Preliminary investigation of the effects of topical mixture of Lawsonia inermis L. and Ricinus communis L. leaves extract in treatment of osteoarthritis using MIA model in rats. Daru 24:12. 10.1186/s40199-016-0152-y 10.1186/s40199-016-0152-y PMC4855329 27142000

