# Supplementary material for: Diminazene aceturate or losartan ameliorates the functional, radiological and histopathological alterations in knee osteoarthritis rodent model: repurposing of the ACE2/Ang1-7/MasR cascade
Source: J Exp Orthop. 2023 Oct 25;10:107. doi: 10.1186/s40634-023-00673-1 (PMC10600085; doi:10.1186/s40634-023-00673-1)
Supplement: Supplementary file 1 — Additional file 1. Shows the principle and procedure of the ELISA assay. [file 40634_2023_673_MOESM1_ESM.docx]

**Principle of the Enzyme-Linked Immunosorbent Assay (ELISA)**

The assay is based on sandwich enzyme-linked immune-sorbent assay technology. Capture antibody was pre-coated onto 96-well plates. The biotin-conjugated antibody was used as a detection antibody. The standards, test samples and biotin-conjugated detection antibody were added to the wells subsequently and washed with wash buffer. After washing, avidin-conjugated Horseradish Peroxidase (HRP) was added, and unbound conjugates were washed away with a wash buffer. TMB substrates were used to visualize the HRP enzymatic reaction. TMB was catalyzed by HRP to produce a blue color product that changed into yellow after adding an acidic stop solution, and the intensity of the color was measured.

**The Assay Procedure**

All samples and standards were assayed in duplicate. 100 μl of standard and sample was added per well, covered with the adhesive strip provided, and incubated. A plate layout was provided to record standards and samples assayed. Then the liquid of each well was removed without washing and 100μl of Biotin-antibody (1x) was added to each well. Then each well was aspirated and washed with wash buffer. Then 100μl of HRP-avidin (1x) was added to each well, covered, and incubated. The aspiration/wash process was repeated five times. Then 90μl of TMB substrate was added to each well. Incubation was made for 15-30 minutes at 37°C. Protection from light was also made. Then 50μl of a stop solution was added to each well. Then finally the optical density of each well was determined within 5 minutes by a microplate reader set to 450 nm.

The results were calculated using the professional soft "Curve Expert” to make a standard curve. The duplicate readings for each sample and standard were averaged and then the average zero standard optical density was subtracted. A best-fit curve was drawn using computer software capable of generating a four-parameter logistic (4-PL) curve fit. The concentration of samples was calculated corresponding to the mean absorbance from the standard curve.
