# Supplementary material for: Diminazene aceturate or losartan ameliorates the functional, radiological and histopathological alterations in knee osteoarthritis rodent model: repurposing of the ACE2/Ang1-7/MasR cascade
Source: J Exp Orthop. 2023 Oct 25;10:107. doi: 10.1186/s40634-023-00673-1 (PMC10600085; doi:10.1186/s40634-023-00673-1)
Supplement: Supplementary file 2 — Additional file 2. Shows the % of change of rotarod performance. [file 40634_2023_673_MOESM2_ESM.docx]

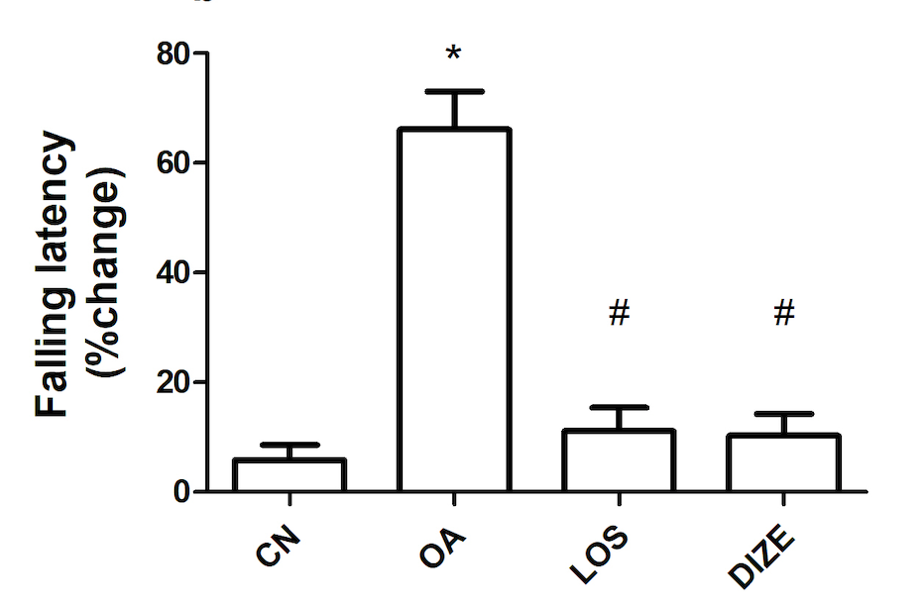


**Evaluation of nociception using tests**. Drugs were administered daily for 21 days. The % of change represents the change after treatment (day 41) compared to day zero. Data are presented as means ± SEM (n=10). Comparisons among groups were analyzed using one-way ANOVA followed by Tukey post-hoc test. Data are compared at *p< 0.05* with CN (*), OA (#). CN; control, OA; MIA induced osteoarthritis, LOS; losartan treated osteoarthritis and DIZE; diminazene treated osteoarthritis.
