# Supplementary figures and images for: Diminazene aceturate or losartan ameliorates the functional, radiological and histopathological alterations in knee osteoarthritis rodent model: repurposing of the ACE2/Ang1-7/MasR cascade
Source: J Exp Orthop. 2023 Oct 25;10:107. doi: 10.1186/s40634-023-00673-1 (PMC10600085; doi:10.1186/s40634-023-00673-1)

**MasR**

**Western Blot bands**


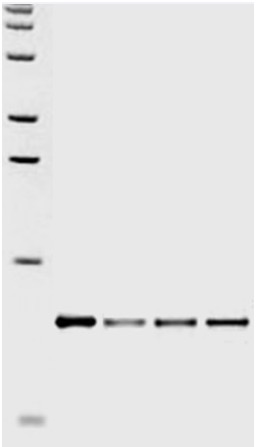


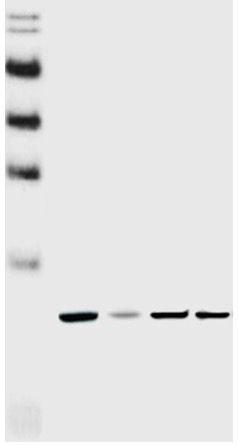

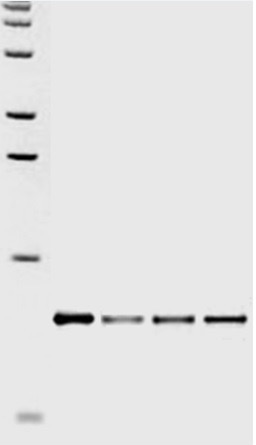


**MMP-13**


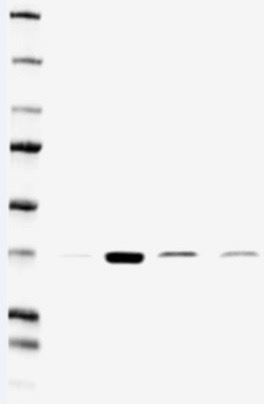


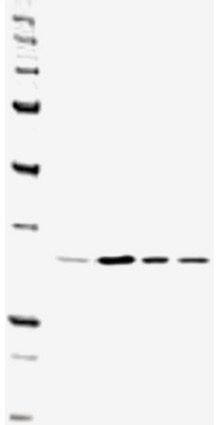

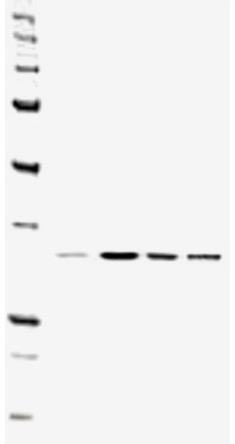


**TNF-α**


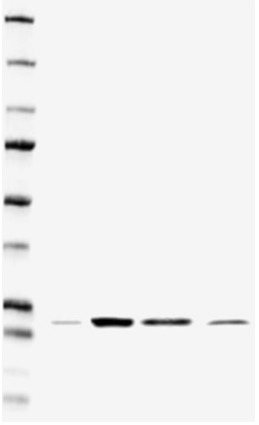

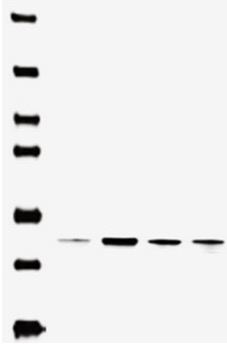


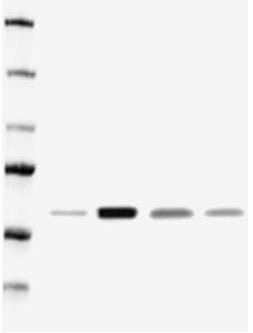


**Beta actin**


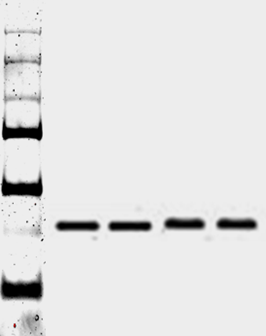

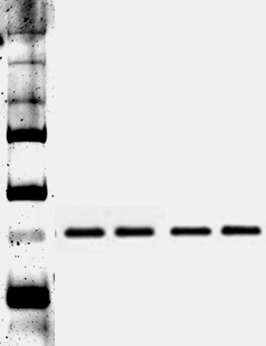

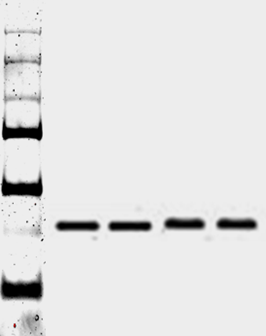

Supplement: Supplementary file 3 — Additional file 3. Shows the original western blot bands. [file 40634_2023_673_MOESM3_ESM.docx]
